# Microbial Enzymes and Their Applications in Industries and Medicine 2014

**DOI:** 10.1155/2015/816419

**Published:** 2015-06-16

**Authors:** Periasamy Anbu, Subash C. B. Gopinath, Bidur Prasad Chaulagain, Thean-Hock Tang, Marimuthu Citartan

**Affiliations:** ^1^Department of Biological Engineering, College of Engineering, Inha University, Incheon 402-751, Republic of Korea; ^2^Institute of Nano Electronic Engineering (INEE) and School of Bioprocess Engineering, Universiti Malaysia Perlis, 01000 Kangar, Perlis, Malaysia; ^3^Department of Microbiology, Molecular Biology and Biotechnology, Himalayan College of Agricultural Sciences and Technology (HICAST), P.O. Box 25535, Kalanki, Kathmandu, Nepal; ^4^Advanced Medical & Dental Institute (AMDI), Universiti Sains Malaysia, 13200 Kepala Batas, Penang, Malaysia

Production of microbial enzymes is a necessary event in the industrial sectors, due to the high and superior performances of enzymes from different microbes, which works well under a wide range of varied physical and chemical conditions. Microbial enzymes serve as a potential replacement, in absence or insufficiency of human enzymes. Further, microbial enzymes are the preferred source of industrial enzymes as they can be produced in large quantities in a short period of time and have shorter generation times, and genetic manipulations can be performed more easily on bacterial cells to increase the enzyme production. Still the industries are looking for new microbial strains in order to produce different enzymes to fulfill the enzyme requirements. This special issue covers ten articles including two review articles, highlighting the importance and applications of biotechnologically relevant microbial enzymes.

In the work carried out by J. R. Wang et al., they have optimized the codon usage in order to enhance the expression level of *α*-amylase gene from* Bacillus licheniformis *in* Pichia pastoris. *A total of 328 nucleotides were altered and the G + C content was improved from 47.6 to 49.2%. The optimized gene has demonstrated a higher level of expression in* P. pastoris *after methanol induction for 168 h in 5- and 50-L bioreactor with the maximum activity of 8100 and 11000 U/mL, compared to the wild type by 2.31- and 2.62-fold. The enzyme is viewed as a potential candidate for *α*-amylase production in industrial use.

L. M. Colla et al. have carried out studies on the partial characterization of lipase acquired from solid-state fermentation by the species of* Aspergillus*. The fungal strains isolated from diesel contaminated soil and selected as the liapse producer. Two types of fermentation were adopted, whereby lipase produced from the submerged fermentation showed an optimum activity at 37°C and pH 7.2. In another fermentation mode, the solid-state fermentation, the optimal temperature and pH for the solid-state fermentation are 35°C and pH 6.0, respectively. Out of the two fermentation modes, lipase produced from the submerged fermentation has more stability at higher temperature than the solid-state fermentation. Lipase from the submerged fermentation still retains 72% of residual activity after one hour of exposure at 90°C. Furthermore, 80% of the residual activity is retained at the acidic pH while lipase obtained from the solid-state fermentation results in residual activity of 60% at the alkaline pH.

N. E.-A. El-Naggar et al. have isolated L-asparaginase producing actinomycete from soil samples. In this study, the isolated strain was identified as* Streptomyces olivaceus* based on the morphological, physiological, biochemical, and molecular analysis. Further, the enzyme production was optimized by response surface method to improve the level of enzyme synthesis. The authors have screened fifteen variables using Plackett-Burman experimental design. The most significant independent variables affecting enzyme production were further optimized by the face-centered central composite design-response surface methodology. Finally, the strain was able to produce a significant level of L-asparaginase enzyme in optimized media.

The review article by F. D. Inácio et al. focused on proteases of wood rot fungi with emphasis on the* Pleurotus* genus. In this review article, the authors have highlighted the characteristics of the* Pleurotus* genus such as easy cultivation techniques, high yield, low nutrient requirements, and its adaptation. In addition, the authors have also highlighted the uses and applications of the proteases in various industries such as biotechnology and medicine.

Another study with amylase is by I. Ali et al.; they performed purification and characterization of polyextremophilic extracellular *α*-amylase from a halophilic strain,* Aspergillus penicillioides,* to be coused with detergents. Purification was performed by ammonium sulfate precipitation and Sephadex G100 gel filtration methods and the purified enzyme has an apparent molecular weight of 42 kDa. The purified enzyme showed higher specificity with the substrate, starch, with an optimal activity at pH 9, 80°C in the presence of NaCl and CaCl_2_. Authors have shown retaining amylase activity as 80% with different laundry detergents, claimed as better activity than commercial amylase.

The review article by S. C. B. Gopinath et al. is on the biotechnological aspects of keratinase production and their future perspectives in industries. Because of ever increasing industrialized food and agriculture market there is high biomass production of the tough biomass keratin which will be biopollutant to the earth and needs to be addressed in a more ecofriendly way. The review highlights the production of keratinase from reliable sources that can be easily managed. The authors put emphasis on microbial keratinase because it is less expensive and more precise than those conventionally used chemicals to treat keratin. The paper discusses different perspective of enzymatic keratinases which can be obtained from fungi, bacteria, and actinomycetes. The attraction to this article for readers is due to the expansion of information on various aspects of keratinase like descriptions on keratinophilic fungi, keratin-degrading bacterial isolates, secretion of microbial keratinases, optimization of keratinase activities, purification process, accelerating microbial keratinase production through biotechnological approach, and developing keratinase sensing technologies in future.

In the article by A. Badhan et al., they have utilized enzyme fingerprinting to identify cell wall components resistant to total tract digestion. Acetyl xylan esterases were identified as the key component to the improved ruminal digestion. Polysaccharide-lignin cross-linked cell wall polymers were identified as the principal components indigested fiber residues in the feces. Enzyme pretreatment was carried out following the analysis of the structural information obtained from the enzymatic fingerprinting and FTIR to enhance glucose yield from barley straw and alfalfa hay. Statistical experimental design was used to analyze the prehydrolysis effects of recombinant acetyl xylan esterases (AXE16B_ASPNG and AXE16A_ASPNG), polygalacturonase (PGA28A_ASPNG), and *α*-arabinofuranosidase (ABF54B_ASPNG) all from* Aspergillus niger*, feruloyl esterase (FAE1a) from* Anaeromyces mucronatus *(expressed in* E. coli*), and endoglucanase GH7 (EGL7A_THITE) from* Thielavia terrestris *(produced in* Aspergillus niger*). Degradation of plant structural polysaccharides is inducted by the fungal fibrinolytic hemicellulases and auxiliary enzymes. Moreover,* in vitro *saccharification of alfalfa and barley straw by mixed rumen enzymes was improved. The model also estimated glucose yield that improves by 75% following pretreatment with polygalacturonase (PGA28A_ASPNG) and *α*-arabinofuranosidase (ABF54B_ASPNG) in 1 : 1 ratio. A hundred percent improvement was obtained after prehydrolysis of barley straw with a mixture of EGL7A_THITE (50%) and FAE1a (50%). Microassay and statistical experimental design can be integrated to predict effective enzyme pretreatments that can enhance plant cell wall digestion by mixed rumen enzymes. This study also has the potential to develop specific enzyme pretreatments for forages, depending on their structure and composition.

B. K. Dash et al. have isolated a new* Bacillus subtilis* strain (BI 19) from the soil and studied the molecular characterizations for the enhanced production of extracellular amylase. In their study, the phylogenetic tree was constructed on the basis of 16S rDNA gene sequences and revealed that this strain clustered with the closest members of* Bacillus* sp. The effect of various fermentation conditions on amylase production from this strain, through shake-flask culture, was investigated. Rice flour was found to be a cheap natural carbon source to induce amylase production. In addition, a combination of peptone and tryptone as organic and ammonium sulfate as inorganic nitrogen sources gave highest yield. Further, maximum production was obtained with the following optimal conditions: 24 h of incubation, 37°C, and pH 8.0. Further increments in the amylase productions were noticed in the presence of Tween 80 and sodium lauryl sulfate. Their results suggest that newly isolated* B. subtilis* BI 19 could be exploited for industrial production of amylase at relatively low cost and time.

S. G. Karp et al. on their original research report discuss the optimization of laccase production and its role in delignification. They have worked on the statistical optimization of laccase production by a strain of* Pleurotus ostreatus* and its delignification properties of sugarcane bagasse. With the determination of the mathematical model of laccase production through the response surface method they have analyzed the role of various factors and came to conclusion that yeast extract as an organic nitrogen source is very important along with appropriate concentration of copper sulfate and ferulic acid and the incubation period in solid-state fermentation for the delignification of sugar bagasse. The authors are successful in reducing the lignin content of sugar bagasse around one-third to one-fourth of the original lignin biomass. Such kind of experiment is important for delignification of biomass for future use in other industrial applications like production of biobased fuel to other organic products like food and beverages to paper and textile industries. The advantages of enzymatic delignification over the conventional physicochemical methods will be less health hazardous to ecosystem in future. The other advantages of laccase enzyme optimization may include relatively higher product yields and fewer side reactions and increased reactor efficiency due to mild reaction conditions and lesser energy requirements.

L. P. Lee et al. have isolated many* Bacillus* species from oil spillage area in Malaysia. In this study, the isolated strains were screened for lipase and protease enzymes using lipid and gelatin, respectively, as the substrates. In addition, the comparison analyses of lipase and protease activities were also analyzed. The result showed that most of the strains were able to produce both enzymes at significant levels. The simultaneous secretion of both the lipase and protease is a means of survival. The isolated* Bacillus* species which harbor lipase and protease enzymes could render potential industrial based applications and solve the environmental problems.

*Periasamy Anbu* *Subash C. B. Gopinath* *Bidur Prasad Chaulagain* *Thean-Hock Tang* *Marimuthu Citartan*

